# 50 years of amino acid hydrophobicity scales: revisiting the capacity for peptide classification

**DOI:** 10.1186/s40659-016-0092-5

**Published:** 2016-07-04

**Authors:** Stefan Simm, Jens Einloft, Oliver Mirus, Enrico Schleiff

**Affiliations:** Department of Biosciences, Molecular Cell Biology of Plants, Goethe University, Max von Laue Str. 9, 60438 Frankfurt/Main, Germany; Molecular Bioinformatics, Cluster of Excellence Frankfurt “Macromolecular Complexes”, Institute of Computer Science, Faculty of Computer Science and Mathematics, Goethe-University Frankfurt, Robert-Mayer-Str. 11-15, 60325 Frankfurt/Main, Germany; Department of Biosciences, Molecular Cell Biology of Plants, Cluster of Excellence Frankfurt (CEF) and Buchmann Institute of Molecular Life Sciences (BMLS), Goethe University, Max von Laue Str. 9, 60438 Frankfurt/Main, Germany

**Keywords:** Hydrophobicity scale, Transmembrane sheets, Transmembrane helix, Beta-sheet, Amino acid pattern, Alternate hydrophobicity

## Abstract

**Background:**

Physicochemical properties are frequently analyzed to characterize protein-sequences of known and unknown function. Especially the hydrophobicity of amino acids is often used for structural prediction or for the detection of membrane associated or embedded β-sheets and α-helices. For this purpose many scales classifying amino acids according to their physicochemical properties have been defined over the past decades. In parallel, several hydrophobicity parameters have been defined for calculation of peptide properties. We analyzed the performance of separating sequence pools using 98 hydrophobicity scales and five different hydrophobicity parameters, namely the overall hydrophobicity, the hydrophobic moment for detection of the α-helical and β-sheet membrane segments, the alternating hydrophobicity and the exact ß-strand score.

**Results:**

Most of the scales are capable of discriminating between transmembrane α-helices and transmembrane β-sheets, but assignment of peptides to pools of soluble peptides of different secondary structures is not achieved at the same quality. The separation capacity as measure of the discrimination between different structural elements is best by using the five different hydrophobicity parameters, but addition of the alternating hydrophobicity does not provide a large benefit. An in silico evolutionary approach shows that scales have limitation in separation capacity with a maximal threshold of 0.6 in general. We observed that scales derived from the evolutionary approach performed best in separating the different peptide pools when values for arginine and tyrosine were largely distinct from the value of glutamate. Finally, the separation of secondary structure pools via hydrophobicity can be supported by specific detectable patterns of four amino acids.

**Conclusion:**

It could be assumed that the quality of separation capacity of a certain scale depends on the spacing of the hydrophobicity value of certain amino acids. Irrespective of the wealth of hydrophobicity scales a scale separating all different kinds of secondary structures or between soluble and transmembrane peptides does not exist reflecting that properties other than hydrophobicity affect secondary structure formation as well. Nevertheless, application of hydrophobicity scales allows distinguishing between peptides with transmembrane α-helices and β-sheets. Furthermore, the overall separation capacity score of 0.6 using different hydrophobicity parameters could be assisted by pattern search on the protein sequence level for specific peptides with a length of four amino acids.

**Electronic supplementary material:**

The online version of this article (doi:10.1186/s40659-016-0092-5) contains supplementary material, which is available to authorized users.

## Background

Hydrophobicity as a physicochemical property is frequently used to characterize secondary structures of proteins. Early on it was noted that this property of amino acids dominates the initial interactions during protein folding [1, 2]. In addition, the physicochemical properties of secondary structures depend on the properties of their amino acids and differ in relation to the native environment of the secondary structure, e.g., in solution or in membranes [3–5]. Considering this, it is not of surprise that the classification and characterization of amino acids according to their hydrophobicity attracted much attention.

In 1962 the first hydrophobicity scale of amino acids was formulated [6]. In addition, a first model to calculate the difference in free energy for the unfolded and native form of the protein catalase in solution was established [6]. Ever since many “hydrophobicity scales” were published. However, not all of these scales focus exclusively on hydrophobicity, but we will continue using this term. The information about hydrophobicity for the amino acids were extracted from biochemical experiments [7], distributions of amino acids in different protein classes [8, 9], the capacity of amino acids to participate in hydrophobic or hydrophilic milieu [10, 11] or from in silico calculations [12]. Today, about 98 “hydrophobicity scales” exist which contain a defined hydrophobicity value for each of the 20 amino acids. A high variance between these scales can be expected due to the variance of the underlying experimental approaches.

At the same time many hydrophobicity parameters for peptide classification have been developed for specific purposes. The overall hydrophobicity was introduced to globally classify peptides [6]. In addition, a hydrophobic moment for detection of the helical membrane segments [13], the alternating hydrophobicity for detection of membrane embedded ß-sheets [14, 15] or exact ß-strand score (EBSS) considering the frequency of amino acids pointing inward or outward of a ß-barrel [16] has been defined.

In parallel many alternative algorithms and methods have been developed to predict protein properties based on hydrophobicity scales and classify them concerning environment (soluble, transmembrane) or function. Among them are routines for the prediction of transmembrane regions [17–20] or protein folding [21–25]. Even today, the hydrophobicity scales are often used to define properties of peptides within proteins [26–29]. However, the wealth of hydrophobicity scales complicates the process of scale selection and of the parameters to be calculated.

Thus, 50 years after formulation of the first scale we analyzed 98 different hydrophobicity scales present in the literature [22, 30, 31]. We used the overall hydrophobicity, the hydrophobic moment for detection of α-helical and β-sheet transmembrane elements, the alternating hydrophobicity and the EBSS as parameters to evaluate their influences on the separation on different secondary structure pools. For the analysis of the different scales and parameters we developed a five dimensional consensus approach to define the quality of the combinatory usage. Finally, we clustered the hydrophobicity scales to classify their performance for general separation capacity of secondary structures, environmental specifications or subsets thereof. We found that the overall performance of the hydrophobicity scales is rather comparable irrespective of the strategy of generation. However, the application of more than one hydrophobicity parameter enhances the capacity of the pool separation, but the alternating hydrophobicity has the lowest impact on the separation capacity when compared with the other four parameters. In general hydrophobicity is suitable to classify transmembrane α-helices and β-sheets better than peptides with other secondary structures. However, specific pattern of four or five amino acids were identified in the different peptide pools analyzed.

## Results and discussion

### Sequence pools, hydrophobicity scales and parameter selection

Different sequence pools were generated to study the separation capacity of hydrophobicity scales and hydrophobicity parameters. To this end, sequences of proteins with known structure were extracted from the ASTRAL40 (http://scop.berkeley.edu/astral/) database [32] and dissected in sequences with exclusive α-helical, ß-sheet and random coil (random) content. The α-helical and ß-sheet sequences were further separated in pools representing transmembrane segments (tm-sheet, tm-helix) and soluble (s-sheet. s-helix; annotated as cytoplasmic). Subsequently, the two small individual transmembrane pools were expanded by one round of psi-blast using the sequences with structural information as bait. Psi-blast for the two small datasets using only sequences of known secondary structures was performed to reach an increase of highly similar sequences from the bait sequence pools. To prevent overfitting of the two pools, filtering for redundant and similar (>95 % sequence identity) sequences was performed. This approach was required to avoid artifacts by comparing drastically different volumes and peptide densities. Otherwise, a small volume could result in an unjustified good separation value. The other three pools (random, s-helix, s-sheet) were not expanded leading to the final sequence number for the five different secondary structure pools (tm-sheet, tm-helix, s-sheet, s-helix, random) (Table 1; Additional file 1: Table S1, Additional file 2: Table S2).

**Table 1:** Sequence pools based on secondary structure dissection

Abbr.	Sequences	Description	Name
random	16447	Non-transmembrane random coils	Random coil
s-sheet	8134	Non-transmembrane β-sheets	Soluble β-sheets
s-helix	34452	Non-transmembrane α-helices	Soluble α-helices
tm-sheet	4407	Transmembrane β-sheets	Trans-membrane β-sheets
tm-helix	9922	Transmembrane α-helices	Trans-membrane α-helices

All peptides have a minimal length of ten amino acids required for EBBS based analysis. Soluble pools contain only peptides with the defined SSE, while the trans-membrane pools contain both, peptides with the given SSE only and with the given SSE and additional amino acids to reach a length of ten amino acids

Further, we implemented an in silico tryptic [K (Lysine)/R (Arginine)] digest of the whole ASTRAL40 [32] database. On the one hand, this approach yields peptides with mixed structural content. On the other hand, on the base of these peptides we wanted to test whether peptides identified by mass spectrometric approaches typically generated by tryptic digest can be used to define topologies of proteins. These sequences were classified according to their continuous (dc-sheet, dc-helix, dc-random) or discontinuous (dd-sheet, dd-helix, dd-random) dominating secondary structure elements (SSE). Peptides without dominating SSE are clustered concerning their portion of different SSE (no-helix, no-sheet, no-random, all). In addition, sequences with transmembrane content were pooled individually for the SSE helix and sheet (krtm-helix, krtm-sheet, Table 2). Combining the pools generated by in silico tryptic digestion or based on known secondary structure approaches, we ended up with 17 different sequence pools. Each of the 17 different sequence pools contains a discrete number of sequences (Tables 1, 2).

**Table 2:** Sequence pools based in silico K/R-digestion

Abbr.	Sequences		Description
dc-helix	8208	Dominating SSE	α-helix; continuous
dc-sheet	1978		β-sheet; continuous
dc-random	4490		random coil; continuous
dd-helix	5154		α-helix; discontinuous
dd-sheet	3322		β-sheet; discontinuous
dd-random	2331		random coil; discontinuous
no-helix	11039	No dominating SSE	β-sheets and random coils
no-sheet	7770		α-helices and random coils
no-random	281		β-sheets and α-helices
all	22237		β-sheets, a-helices, and random coils
krtm-helix	214		TM α-helices with additional AA at N- or C-terminus
krtm-sheet	119		TM β-sheets with additional AA at N- or C-terminus

All peptides have a minimal length of ten amino acids (AA). Dominating SSE stands for SSE content larger than 70 % of all AA. Continuous means that this SSE is without gap

*TM* transmembrane. The nomenclature is as follows: *dc* peptide derived by in silico tryptic digest with continuous structural element, *dd* peptide derived by in silico tryptic digest with discontinuous structural element, *krtm* peptide derived by in silico tryptic digest (kr) with transmembrane segment

After gathering the test pools different hydrophobicity scales were extracted from literature (Tables 3, 4; Additional file 1: Table S1). The 98 selected scales cover experimentally developed scales, calculated scales as well as scales which have been created by improving pre-existing scales. In addition, eight scales represent the reverse algebraic numeral scales to other scales to test if the algebraic numeral has an influence on the results.

**Table 3:** Improved, calculated and inverted hydrophobicity scales

	ID	Year	Author	Scale	Ref.
Improved	65	1968	Zimmerman	ZIMJ680101	[36]
	54	1971	Tanford and Nozaki	NOZY710101	[37]
	29	1975	Jones	JONES	[35]
	63	1975	Jones	JOND750101	[35]
	78	1976	Levitt	LEVIT	[44]
	27	1982	Kyte and Doolittle	KYTJ820101	[38]
	12	1983	Sweet and Eisenberg	SWER830101	[42]
	83	1983	Sweet and Eisenberg	SWEET	[42]
	3	1984	Eisenberg	ESID840101	[45]
	51	1984	Eisenberg	EISEN	[45]
	47	1985	Guy	GUYH850101	[46]
	74	1985	Guy	GUYFE	[46]
	80	1985	Rose	ROSEF	[47]
	57	1985	Rose	ROSG850102	[47]
	82	1987	Cornette	NNEIG	[41]
	41	1989	Cohen and Kuntz	COHEN	[48]
	44	1998	Juretic	MDK0	[22]
	45	1998	Juretic	MDK1	[22]
	24	1998	Juretic	JURD980101	[22]
	89	2005	Zviling	SET1	[12]
	90	2005	Zviling	SET2	[12]
	91	2005	Zviling	SET3	[12]
Calculated	38	1976	Chothia	CHOTA	[49]
	50	1976	Chothia	CHOC760103	[49]
	75	1976	Chothia	CHOTH	[49]
	72	1980	Ponnuswamy	PONNU	[8]
	81	1983	Sweet and Eisenberg	Sweet and Eisenberg	[41]
	36	1985	Kidera	KIDER	[50]
	39	1985	Rose	ROSEB	[47]
	55	1985	Welling	Welling	[51]
	53	1986	Rao and Argos	Rao and Argos	[52]
	70	1989	Fasman	FASG890101	[48]
	79	1989	Fasman	GIBRA	[48]
Inverted	92	1973	Bull and Breese	BULDG reverse	[53]
	64	1976	Levitt	LEVM760101 reverse	[54]
	95	2001	Bishop	Bishop reverse	[55]
	59	1996	Wimley and White	Wimley reverse	[56]
	94	1985	Welling	Welling reverse	[51]
	26	1986	Engelman	ENGD860101 reverse	[39]
	40	1985	Rose	ROSEA reverse	[47]
	21	1995	Wilce	WILM950103 reverse	[7]
	23	1995	Kuhn	KUHL950101 reverse	[57]
	11	1990	Prabhakaran	PRAM900101 reverse	[58]
	96	1989	Fasman	FASG890101 reverse	[48]
	93	1985	Guy	GUYH850101 reverse	[46]
	25	1985	Kidera	KIDA850101 reverse	[50]
	98	1988	Roseman	ROSM880102 reverse	[59]
	88	1988	Roseman	ROSM880103 reverse	[59]
	97	1988	Roseman	ROSM880101 reverse	[59]
	84	1981	Wolfenden	WOLR810101 reverse	[60]

Shown are the category of the scale (column 1), the ID of the hydrophobicity scale (column 2) the year of the publishing (column 3), the name of the authors (column 4) and the name of the scale (column 5)

**Table 4:** Experimental hydrophobicity scales

ID	Year	Author	Scale	Ref.
46	1973	Bull and Breese	BULDG	[53]
4	1978	Manavalan and Ponnuswamy	MANP780101	[61]
76	1979	Heijne and Bloomberg	VHEG790101	[62]
52	1979	Janin	JANJ790102	[63]
73	1979	Janin	JANIN	[63]
37	1979	Wolfenden and Cullis	WOLR790101	[60]
5	1980	Ponnuswamy	PONP800101	[8]
6	1980	Ponnuswamy	PONP800102	[8]
7	1980	Ponnuswamy	PONP800103	[8]
8	1980	Ponnuswamy	PONP800104	[8]
9	1980	Ponnuswamy	PONP800105	[8]
10	1980	Ponnuswamy	PONP800106	[8]
49	1981	Wilson	Wilson	[64]
61	1982	Argos	ARGP820101	[65]
62	1983	Fauchere and Pliska	FAUJ830101	[11]
71	1983	Fauchere and Pliska	FAUCH	[11]
48	1985	Miyazawa and Jerningen	MIYS850101	[66]
28	1986	Engelman	ENGEL	[39]
77	1988	Roseman	ROSEM	[59]
42	1989	Jacobs and White	JACWH	[67]
56	1990	Parker	PARJ860101	[68]
85	1990	Cowan and Whittacker	Cowan Whittacker	[59]
86	1988	Roseman	ROSM880101	[59]
87	1988	Roseman	ROSM880102	[59]
68	1990	Cowan and Whittacker	COWR900101	[69]
69	1991	Black and Mould	BLAS910101	[70]
43	1992	Cassari and Sippl	CASSI	[71]
1	1992	Cid	CIDH920101	[9]
2	1992	Cid	CIDH920105	[9]
30	1992	Cid	CIDBB	[9]
31	1992	Cid	CIDA+	[9]
32	1992	Cid	CIDAB	[9]
33	1993	Ponnuswamy and Gromiha	PONG1	[72]
34	1993	Ponnuswamy and Gromiha	PONG2	[72]
35	1993	Ponnuswamy and Gromiha	PONG3	[72]
67	1993	Ponnuswamy and Gromiha	PONP930101	[73]
19	1995	Wilce	WILM950101	[7]
20	1995	Wilce	WILM950102	[7]
22	1995	Wilce	WILM950104	[7]
60	1996	Wimley and White	Wimley	[56]
58	2001	Bishop	Bishop	[55]
13	2001	Naderi-Manesh	NADH010101	[40]
14	2001	Naderi-Manesh	NADH010102	[40]
15	2001	Naderi-Manesh	NADH010103	[40]
16	2001	Naderi-Manesh	NADH010104	[40]
17	2001	Naderi-Manesh	NADH010106	[40]
18	2001	Naderi-Manesh	NADH010107	[40]
66	2001	Naderi-Manesh	NADH010105	[40]

Shown are the ID of the hydrophobicity scale (column 1), the year of the publishing (column 2), the name of the authors (column 3) the name of the scale (column 4) and a relevant reference the scale was extracted from

To investigate the separation capacity of the selected hydrophobicity scales on the different defined sequence pools five measurement parameters have been defined (Table 5). The EBSS [16], the alternating hydrophobicity [14, 15] and the hydrophobic moment calculated for β-sheets with an angle of 180° [13] are hallmarks for hydrophobic content, while the hydrophobic moment calculated for α-helices [14] and the average hydrophobicity [33] are used to identify α-helical transmembrane regions (hydrophobic-moment α). Each of the parameters was calculated in a sliding window of ten amino acids. The largest and smallest value for each peptide was considered as outlined below.

**Table 5:** Hydrophobicity parameters

Index	Name	Description
0	Max. exact β-strand score (EBSS)	Parameter to score the probability of a sequence with ≥10 AA to be a TM β-sheet [16]
1	Min. exact β-strand score (EBSS)	
2	Max. alternating hydrophobicity	High alternating hydrophobicity probing for polar and unpolar AA alternation typical for transmembrane β-sheets [14, 15]
3	Min. alternating hydrophobicity	
4	Max. hydrophobicity-moment α	Analyzing the distribution of hydrophobicity considering the amino acid distribution in α-helices with an angle between amino acids of 100° to probe for potential to form a TM α-helix [13]
5	Min. hydrophobicity-moment α	
6	Max. hydrophobicity-moment β	Analyzing the distribution of hydrophobicity considering the amino acid distribution of β-sheets with an angle between amino acids of 180° to probe for potential to form a TM β-sheet [13]
7	Min. hydrophobicity-Moment β	
8	Max. average hydrophobicity	Average hydrophobicity of the peptide [33]
9	Min. average hydrophobicity	

Shown are the index (column 1), the name (column 2) and the description of the used hydrophobicity parameters (column 3). The description is equal for the minimum and maximum of the used hydrophobicity parameter

### The relation of the hydrophobicity scales

**Fig. 1:**
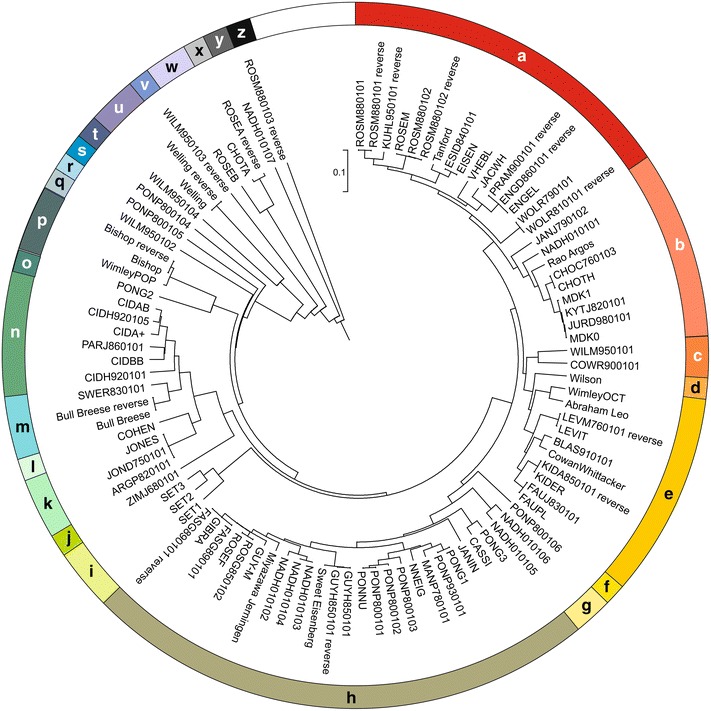
Clustering of hydrophobicity scales. Shown is the UPGMA tree of the clustered hydrophobicity scales based on the normalized amino acid value distances (see “Methods” section). The hydrophobicity scales are clustered to groups (*a* to *z*) within similarity larger than 0.07

We clustered the hydrophobicity scales by comparing the hydrophobicity value for each amino acid to each other. The variance of the different hydrophobicity scales was analyzed by Pearson correlation. The values obtained were used to calculate the dissimilarity ($= \sqrt { 1\,{ - }\,{\text{correlation}}^{2} }$) to create an Unweighted Pair Group Method with Arithmetic mean (UPGMA) tree of the hydrophobicity scales via MEGA6 [34]. The tree was used to cluster those scales to groups of similar amino acid value behavior setting a threshold (Fig. 1; Additional file 2: Table S2). The linearized UPGMA tree of the 98 hydrophobicity scales was inspected to split the scales in clusters using a threshold of a maximal dissimilarity of 0.05. The created clusters were named alphabetically. The UPGMA tree was circled to give an overview of all 98 hydrophobicity scales and their position at a glance (Fig. 1).

As expected, the hydrophobicity scales generated by inverting the amino acid values cluster with the original scales (Table 3). However, not all hydrophobicity scales that have been created by the same experimental approach or by the same author cluster together (Table 4). Most prominent examples are (i) the scales generated by Jones (scales 29, 63; JONES, JOND750101; cluster K) [35] which adjusted the scale of Zimmerman (scale 69; ZIMJ680101; cluster J) [36] by considering experimental derived values (scale 54, Tanford; cluster A) [37]; or (ii) the scales proposed by Zviling (scales 89-91; SET1-3; cluster I) [12] that have been based on the scales of Kyte and Doolittle (cluster B) [38] and Engelman (cluster A) [39].

### Calculation of separation capacity of hydrophobicity scales

Next we analyzed the capacity of the 98 hydrophobicity scales to separate the 17 defined sequence pools. The initial analysis was based on all five hydrophobicity parameters. For each parameter the maximal and minimal value for each peptide was calculated (Table 5). However, we realized that the simultaneous application of the minimal and maximal value of the same parameter does not increase the separation performance. By this we limit the parameter selection too either the minimal or the maximum value. We calculated the 32 parameter combinations (five parameter and alternating minimal and maximal value) for each peptide for all 98 hydrophobicity scales. The resulting five dimensional vectors for each peptide and each hydrophobicity scale were used to define five dimensional clouds for each pool and each specific hydrophobicity scale.

**Fig. 2:**
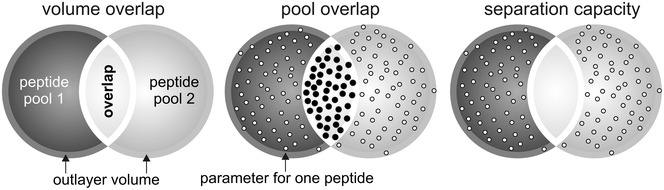
Scoring scheme. Shown is the scoring scheme of the separation of two sequence pools. Both sequence pools (*dark grey* area, *grey* area) build a cloud. The overlap of both clouds (*light grey* area) as well as the sequences (*bold* points) in this overlap is used to calculate the separation capacity. The *circle* surrounding both clouds represents the volume of the outlayer

For the analysis of the separation capacity (Fig. 2, right) of a scale between two clouds of sequence pools we calculated the overlap volume (Fig. 2, left) and the number of peptides within the overlap (Fig. 2, middle). The size of the overlap volume and the number of peptides within the overlap volume negatively correlates with the separation capacity for two sequence pools. Further, we defined the “convex envelope” as described in the “Methods” section. Next we removed all peptides, which are part of the convex envelope. We recalculated the volumes of the pools and repeated the last step of the routine with the new volumes. Removing the peptides on the convex envelope was performed because the presence of only few peptides positioned distantly from the other peptides could increase the volume drastically. In case that the peptides are positioned close to others the volume did not change significantly and thus, this step improved the reliability of assignment of the majority of peptides.

Next, we defined a separation capacity score (Formula 1) to rank all scenarios. S_v_ is a score based on the volume of the overlap in relation to the volumes of the two clouds. For S_p_ we counted all peptides in the overlap volume of two sequence pools and set them in relation to all peptides of the two clouds. The score S is scaled between zero (both clouds totally overlap) and one (no peptides in overlap volume) and gives the quality of a certain hydrophobicity scale for the separation of two defined pools.

#### Formula 1

Separation capacity score

$$\begin{aligned} S &= S_{v} *S_{p} ;\quad S_{v} = 1 - \left( {2* \frac{{V_{ov} }}{{(V_{1} + V_{2} )}}} \right) ;\\ S_{p} &= 1 - \left( {\frac{{P_{ov} }}{{(P_{1} + P_{2} )}}} \right) \end{aligned}$$

Here, P_1_ and P_2_ are the total numbers of peptides of pool 1 and 2, P_*ov*_ is the number of all sequences in the overlap volume, V_1_ and V_2_ are the volumes defined by the sequence pools 1 and 2, and V_*ov*_ is the overlapping volume of both pools. The number of V_i_ and P_i_ was always i = 2 because two pools were analyzed in parallel.

**Fig. 3:**
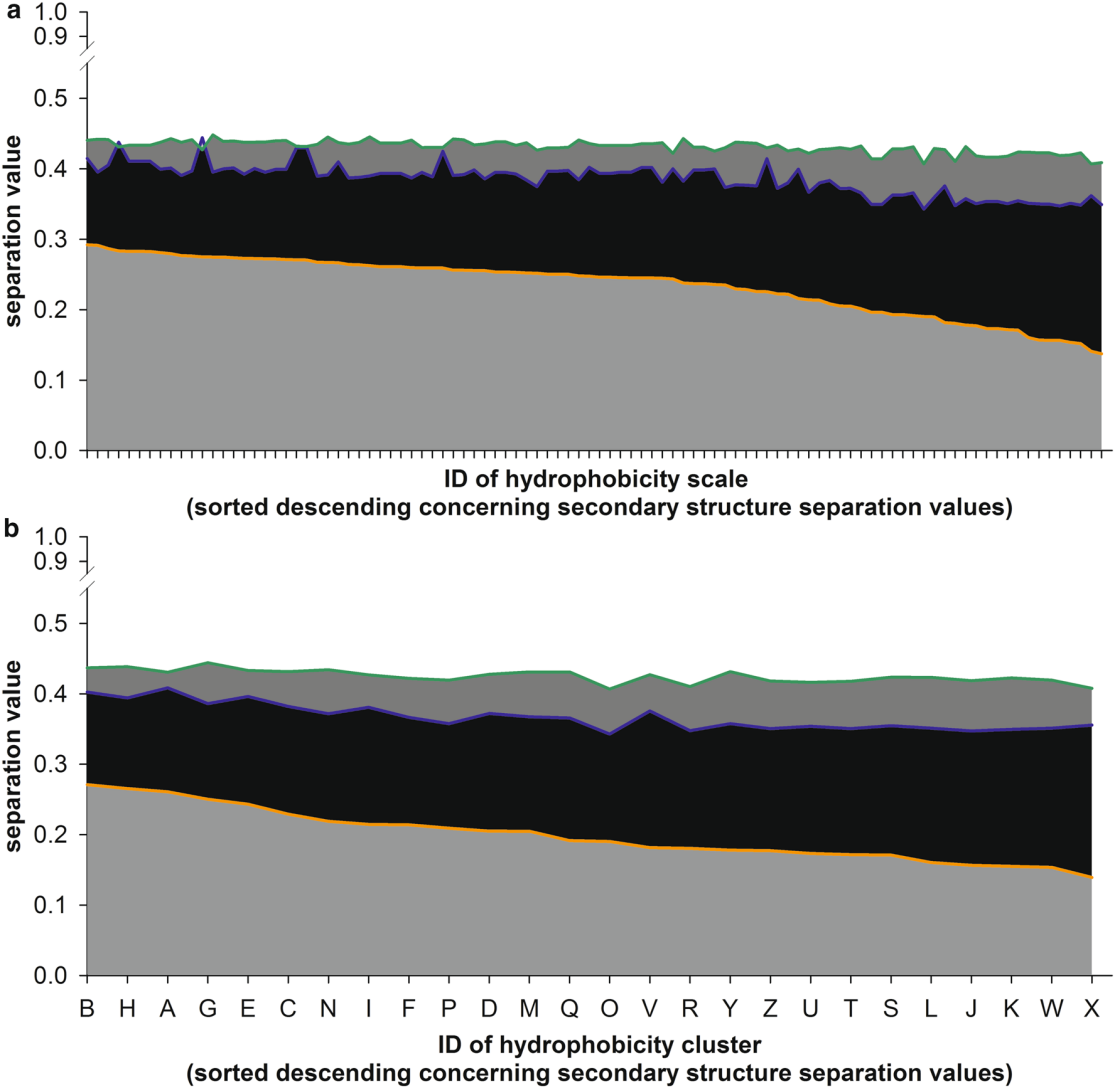
Separation of pools by hydrophobicity scales. **a** Shown is the overall separation value for each hydrophobicity scale for the secondary structure (*orange*), in silico tryptic digest (*blue*) and mixed (*green*) sequence pools as area plot. The hydrophobicity scales are sorted from highest to lowest value. **b** The same as in **a** but the separation value is calculated for the cluster of hydrophobicity scales

The general S value was calculated for each scale for the sequence pools based on secondary structure dissection (Table 1; Fig. 3a, orange line), the sequence pools generated by in silico digestion (Table 2; Fig. 3a, blue line) and both together, the sequence pools based on secondary structure dissection and the sequence pools generated by in silico digestion (Fig. 3a, green line: mixed). While all scales perform similar for the pool derived by digestion or by combining all pools, a distinction is found for the “structure” pool. The three best performing hydrophobicity scales are 14, 15 (NADH010102, NADH010103; Table 4) [40] and 82 (NNEIG; Table 3) [41]. Scales 14 and 15 are experimental hydropathic scales and based on self-information values of a two state model with 9 and 16 % protein surface accessibility. Scale 82 is an improvement of the scale of Sweet and Eisenberg from 1983 [42] using the eigenvalues of a normalized nearest neighbor matrix.

In parallel, the average S value for the clusters of hydrophobicity scales (Fig. 1) defined according to the UPGMA-tree was calculated (Fig. 3b). The average S values observed for the peptide pools obtained by in silico tryptic digest (Fig. 3b, blue) or by the combination of peptide pools generated by the two strategies (Fig. 3b green) do not show a dependence on the selected cluster. Only for the secondary structure peptide pools the observed average S values differ between 0.28 for the cluster B and 0.13 for cluster X. Moreover, after sorting the clusters according to the average S values for the secondary structure peptide pools the order of clusters does not follow the order in the UPGMA tree (Fig. 3b, orange).

### Separation of specific structure pools via hydrophobicity

**Fig. 4:**
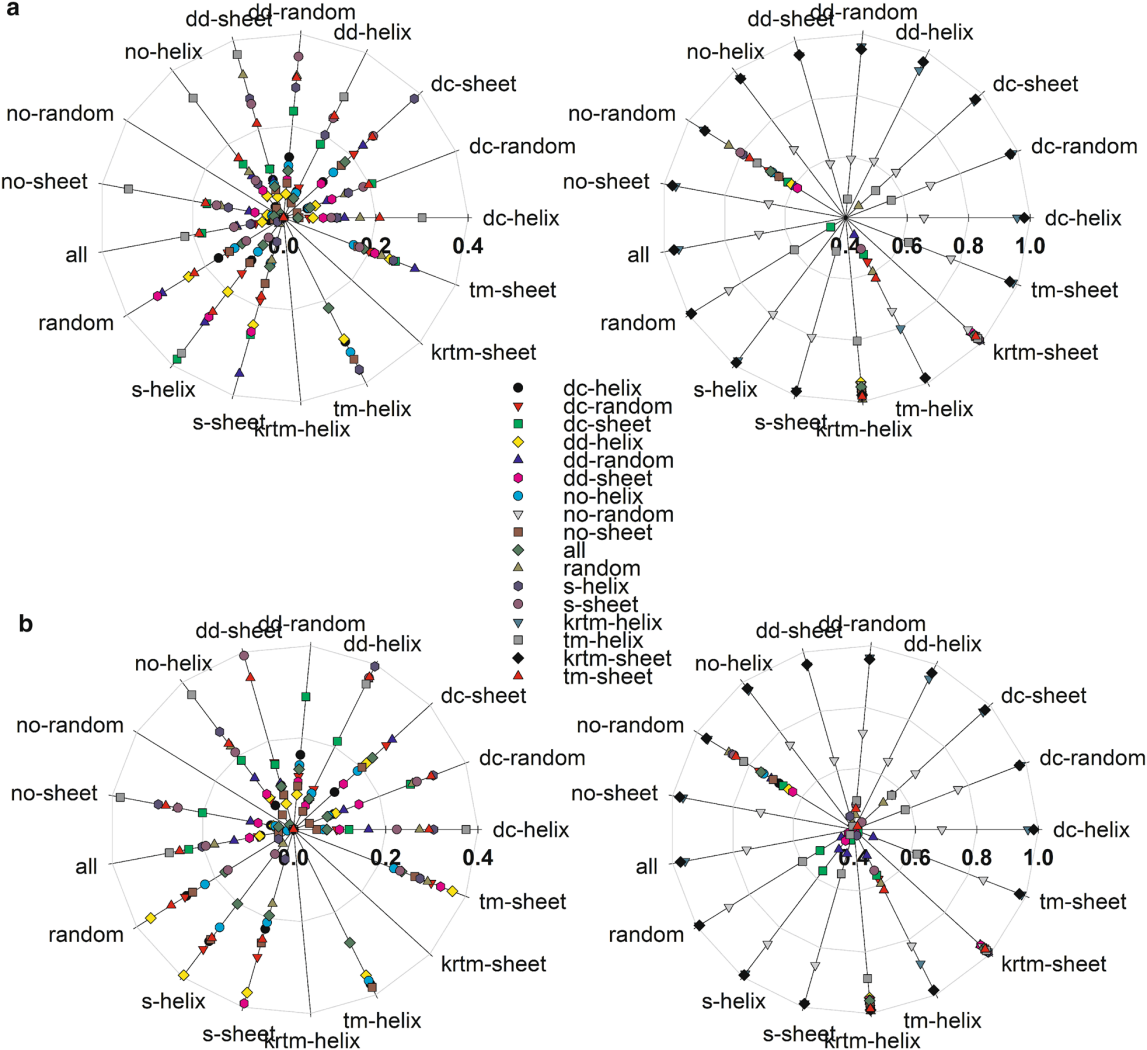
Separation capacity of specific sequence pools. Shown is the pairwise separation capacity for the scale 14 (**a**) and for the best value of any of all hydrophobicity scales as radar plot (**b**) focusing on separation capacity below 0.4 (*left*) and above 0.4 (*right*). Each *line* represents one pool, at which the separation to all other pools is represented by the according *symbol*

The moderate separation capacity of all hydrophobicity scales using all 17 sequence pools prompted us to inspect the separation of the individual sequence pool pairs. The results are exemplified for the separation value for the best performing scale 14 [40] (Fig. 4a) as well as the maximal separation capacity out of all 98 hydrophobicity scales for each pairwise sequence pool combination (Fig. 4b). Globally, the S values obtained for the pairwise pool separation by maximal separation capacity out of all 98 hydrophobicity scales (Fig. 4b) are in general larger than the S values obtained by using the best performing scale 14 only.

In detail, the three pools with transmembrane α-helix (krtm-helix), with transmembrane β-sheet (krtm-sheet) or without random coil content (no-random) generated by digestion have the largest S value while analyzing the overlap with other sequence pools, irrespective whether the best scale (Fig. 4a) or the best value (Fig. 4b) is considered. In contrast, the secondary structure transmembrane pools (tm-sheet, tm-helix) show low S values while analyzing the overlap with other pools. Nevertheless, the S values of the secondary structure transmembrane pools are larger than the S values found while analyzing the overlap of the remaining sequence pools (Fig. 4b).

Remarkably, high S values were found when the overlap between the two secondary structural transmembrane pools (tm-sheet, tm-helix) and the three pools with transmembrane α-helix (krtm-helix), with transmembrane β-sheet (krtm-sheet) or without random coil content (no-random) generated by digestion was calculated. This might suggest that the regions flanking the transmembrane domain present in the sequences of the peptide pools generated by digestion provide additional information. This information in combination with the hydrophobicity might give an additional signature for such domains. Hence, in silico digestion with subsequent analysis by the described parameters using e.g. the hydrophobicity scale 14 can be used to detect transmembrane helices and sheets.

With respect to the remaining pools we observed that the S value obtained while analyzing the overlap of the secondary structure pools (s-sheet, s-helix and random) is higher when compared to the pools containing sequences with mixed structures (Fig. 4a, b). This result is expected, as the chosen parameters detect the individual elements and a mixture thereof yields mixed information.

**Fig. 5:**
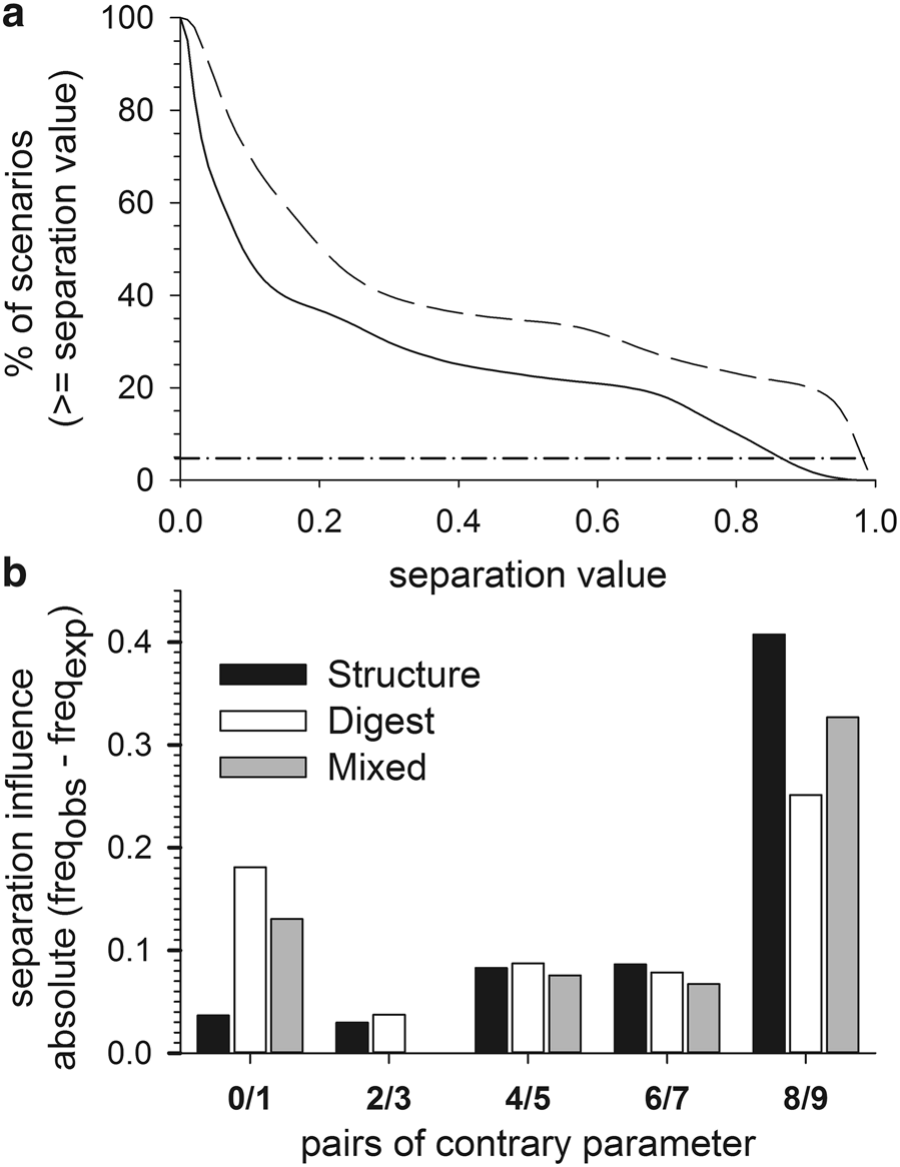
Influence of hydrophobicity parameter for separation. **a** Shown is the percentage of scenarios reaching a specific separation values for all sequence pools including outliers (*dashed line*) and without outliers (*solid line*). The *dash-dotted line* shows the best separated 5 % of all scenarios and serves as marginal value to detect the threshold for analyzing the influence of the different hydrophobicity parameter to the separation. **b** Shown is the influence on separation of the ten hydrophobicity parameters (Table 5) for the secondary structure based sequence pools (*black*), the sequence pools generated by digestion (*white*) and the combination of both (*grey*). The hydrophobicity parameters are paired (max., min.). The separation influence is calculated as absolute value of the difference between observed and expected frequency of the best 5 % of separated scenarios (Fig. 5a)

Consequently, we analyzed the performance of the individual hydrophobicity parameters and the usage of the multi-dimensional approach. We realized that the S value is more dependent on the combination of the hydrophobicity parameter in a multi-dimensional vector than on a specific hydrophobicity scale (Additional file 3: Fig. S1). Including the top 5 % of all scenarios for separating two pools from each other (Fig. 5a) the influence of the different parameter varies significantly (Fig. 5b). It becomes obvious that the average hydrophobicity and EBSS have the strongest impact on the separation quality, while the alternating hydrophobicity previously thought to specifically recognize transmembrane β-strands [14, 15] has the lowest impact on separation. Only for the mixed situation we observed that the alternating hydrophobicity has no impact at all. Thus, the overall performance is dependent on all parameter although to a different extent.

### Benefit of amino acid pattern to separate specific structure pools

An amino acid based approach for the different structure pools was subsequently considered in addition to the hydrophobicity based separation. At first the amino acid composition of the different pools was analyzed, which did not yield a significant difference between the individual pools (Additional file 4: Table S3).

**Fig. 6:**
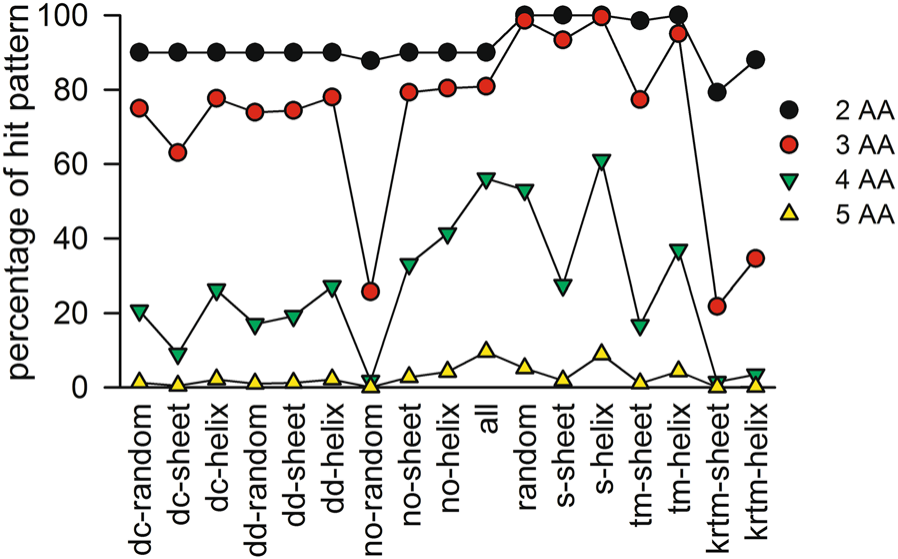
Amino acid pattern distribution. Shown is the percentage of occurrence of all possible amino acid pattern of a specific length in the different sequence pools. The length of the pattern varies from 2 to 5. 2 AA *black circle*; 3 AA *red circle*; 4 AA *green triangle down*; 5 AA *yellow triangle up*

Thus, the occurrence of amino acid patterns of two to five amino acids was analyzed utilizing a markov chain approach (Fig. 6; “Methods” section). Nearly all (~80 %) detected amino acid patterns up to the length of three occur in each of the different sequence pools. The number of globally occurring amino acid patterns of a length of four drops down to 60 %. An elongation of the pattern length to more than five amino acids results in coverage of less than 5 % and thus, is not of use for separation. Thus, the exclusive appearance or at least overrepresentation of amino acid patterns of four and five amino acids in the distinct sequence pools was analyzed.

We identified several peptides of four (Table 6; Additional file 5: Table S4) and five amino acids that are specific or at least enriched in peptides of a certain pool (Table 7; Additional file 6: Table S5). Analyzing the frequency of occurrence of these peptides revealed that patterns with four amino acids are only very moderately specific as only very few are at least 50-fold more frequent in a certain pool than in general (Table 6). In turn, patterns with five amino acids can serve as an additional discriminator because patterns with 500-fold higher frequency in a specific pool than in the overall sequence pool exist (Table 7). This holds true particularly when the sequence pools generated by the same strategy (Tables 1, 2) are compared. This information can only be taken in addition to the hydrophobicity parameters, because occurrence of specific amino acid patterns in one specific structure pool compared to all pools as reference did not yield an adjusted p value below 0.05 for any amino acid pattern of length five. Nevertheless, detection of ß-strands in peptides can be supported by the detection of penta-peptides YLVNM (dc-sheet), LTVTG, TLDGG (dd-sheet), CGGSL and YGGVT (s-sheet). Remarkably, the penta-peptides observed for the structurally derived pool is not overlapping with the penta-peptides observed for the pool derived by in silico *tryptic* digestion which might suggest that the latter contain specific regions at the end of the strand. Moreover, amino acid patterns specific for the transmembrane ß-strands are SIGA (krtm-sheet, Table 6), LYGKV, PTLDL and SASAG (tm-sheet, Table 7). For peptides with mainly random content we found that S-GSSG-S, SGPSS or TILPL are enriched (random, dd-random; Table 7). In turn, for pools mainly consisting of helical structures we found only one penta-peptide specific pattern for the structural pools with α-helix (s-helix; EELKK) and for the pool of peptides with the transmembrane α-helix (krtm-helix; YVFFG; Table 7). Thus, a prescreening of sequence pools with these amino acid patterns might improve the classification quality.

**Table 6:** Patterns of four amino acids

Sequence pool	Peptide	FO in pool	Average FO in remaining set	Average FO in remaining set GBSS
dc-helix	ALAA	0.0005088	0.0001336	0.0001327
	AALA	0.0004970	0.0001545	0.0001559
	ALLE	0.0003787	6.55e−05	6.82e−05
dc-random	GSSG	0.0028090	0.0002055	0.0001760
	SSGS	0.0015990	0.0001405	0.0001205
	SGSS	0.0014350	0.0001022	7.90e−05
	HHHH	0.0007792	4.17e−05	2.41e−05
	EEEE	0.0003691	2.28e−05	1.88e−05
dc-sheet	VLLV	0.0003481	0.0001082	0.0001198
dd-helix	EELL*	*0.0003622*	*5.37e−05*	*5.67e−05*
	LEEL*	*0.0003372*	*6.47e−05*	*6.58e−05*
dd-random	GSSG	0.0006914	0.0003379	0.0003685
	SSGS	0.0003872	0.0002163	0.0002307
	SGSS	0.0003042	0.0001729	0.0001818
	SSGL	0.0002489	4.35e−05	4.62e−05
dd-sheet	GEVV*	*0.0002647*	*4.57e−05*	*4.93e−05*
	PDGT*	*0.0002427*	*1.19e−05*	*1.53e−05*
	DGSV*	*0.0002427*	*2.88e−05*	*3.05e−05*
no-helix	SGSS	0.0001950	0.0001797	0.0001918
	PDGS	0.0001509	2.59e−05	3.24e−05
no-random	VVGI	0.0017010	4.14e−05	3.84e−05
	QELD	0.0010210	*1.13e*−*05*	*1.45e*−*05*
no-sheet	LEAL	0.0003080	8.56e−05	9.38e−05
random	GSSG*	*0.0012510*	*0.0003029*	*2.53e−05*
	GPSS	0.0007403	0.0001942	4.57e−05
	SGPS	0.0006586	0.0001508	2.70e−05
	SSGS	0.0005360	1.43e−05	*6.00e*−*07*
	SGSS	0.0005156	1.14e−05	*3.60e*−*06*
s-sheet	VKVI	0.0001704	2.9e−06	1.16e−05
krtm-helix	LGLL	0.0012460	6.78e−05	5.58e−05
	VLLV	0.0009341	7.16e−05	6.66e−05
	GIAL	0.0009341	5.29e−05	4.11e−05
tm-helix	LLLL	0.0004439	7.87e−05	4.07e−05
	LILL	0.0004040	3.21e−05	1.25e−05
	LLLV	0.0003990	4.38e−05	2.15e−05
	ILLL	0.0003891	2.98e−05	1.73e−05
krtm-sheet	SIGA	0.0012020	2.92e−05	*1.69e*−*05*
tm-sheet	SGPL	0.0004559	1.88e−05	1.20e−05
	SLNL	0.0004079	1.96e−05	2.15e−05
	LYGG	0.0004079	1.06e−05	9.80e−06

Given are the sequence pool name (column 1) and peptide sequence with the highest frequency of occurrence (column 2); the frequency of occurrence (FO) of this peptide in the according pool (column 3), the frequency of occurrence of this peptide in the pool containing all sequences except the one of the analyzed pool (column 4), the frequency of occurrence of this peptide in the pool containing all sequences generated by the same strategy (GBSS) as the analyzed pool excluding the sequences of the analyzed pool (column 5). Italic shows peptides that have an at least 50-fold higher frequency, with respect to the remaining sets (column 4) or the remaining peptides generated by the same strategy (column 5). Peptide sequences with p values below 0.05 were marked by an asterisk

**Table 7:** Amino acid patterns of five amino acids

Sequence pool	Peptide	FO in pool	FO in remaining set	FO in remaining set GBSS
dc-helix	ALLDA	0.0001311	5.50e−06	3.50e−06
	AALAA	0.0001311	3.75e−05	4.50e−05
	ALDAA	0.0001180	6.30e−06	5.50e−06
	AAALA	0.0001180	1.31e−05	1.15e−05
dc-random	GSSGS	0.0016490	9.41e−05	7.02e−05
	SGSSG	0.0015360	8.46e−05	6.15e−05
	SSGSS	0.0015130	8.75e−05	6.31e−05
	HHHHH	0.0005872	2.50e−05	1.33e−05
dc-sheet	VLVNA	0.0001966	4.80e−06	*1.10e*−*06*
	SDTVV	0.0001966	*2.50e*−*06*	*3.20e*−*06*
	KGTVT	0.0001966	*2.50e*−*06*	*2.20e*−*06*
	YLVNM^#^	0.0001311	<*1.00e*−*08*	<*1.00e*−*08*
dd-helix	LTEEE	8.01e−05	2.10e−06	2.10e−06
	LTLEE	9.34e−05	5.20e−06	7.30e−06
	ELLAD	8.01e−05	7.50e−06	9.20e−06
dd-random	GSSGS	0.0003547	0.0001750	0.0001879
	SGSSG	0.0003252	0.0001603	0.0001716
	SSGSS	0.0003252	0.0001618	0.0001711
	GSSGL	0.0001774	1.28e−05	1.35e−05
	TILPL^#^	0.0001182	*1.20e*−*06*	*2.00e*−*07*
dd-sheet	TLDGG^#^	0.0001182	4.50e−06	*2.00e*−*07*
	SVIDT	9.52e−05	*1.40e*−*06*	2.10e−06
	LTVTG^#^	9.52e−05	2.80e−06	*2.00e*−*07*
no-helix	GDSGG	6.76e−05	2.20e−06	3.20e−06
no-random	VGIVT^#^	0.0011290	*5.00e*−*07*	*2.00e*−*07*
	TGHSL^#^	0.0007524	*1.20e*−*06*	*1.70e*−*06*
no-sheet	SSGSS	0.0002080	0.0001691	0.0001817
	SGSSG	0.0001976	0.0001683	0.0001832
all	VIGGG	4.35e−05	3.90e−06	2.70e−06
	IIGGG	3.84e−05	2.50e−06	2.70e−06
	LADAG	3.07e−05	2.00e−06	2.40e−06
	IVGAG	3.07e−05	4.80e−06	6.00e−06
	GVDVV	3.07e−05	1.20e−06	1.20e−06
random	SGPSS^#^	0.0005350	*2.00e*−*07*	<*1.00e*−*08*
	GSSGS^#^	0.0007301	0.0001515	*7.00e*−*07*
	SGSSG^#^	0.0006744	0.0001385	*7.00e*−*07*
	SSGSS	0.0006744	0.0001399	*8.00e*−*06*
	HHHHH	0.0002508	4.60e−05	*7.00e*−*07*
s-helix	EELKK^#^	5.49e−05	<*1.00e*−*08*	<*1.00e*−*08*
s-sheet	CGGSL^#^	0.0001124	6.60e−06	<*1.00e*−*08*
	GIVSW	8.03e−05	4.80e−06	*1.30e*−*06*
	YGGVT^#^	6.42e−05	1.01e−05	<*1.00e*−*08*
krtm-helix	LLVGI	0.0004832	*4.60e*−*06*	*2.60e*−*06*
	LAAVA	0.0004832	*7.20e*−*06*	*3.10e*−*06*
	FLAVL	0.0004832	*3.00e*−*06*	*1.20e*−*06*
	YVFFG^#^	0.0003221	*7.00e*−*07*	<*1.00e*−*08*
	YPIVW	0.0003221	*5.40e*−*06*	*6.00e*−*06*
tm-helix	LILLL	9.97e−05	*1.00e*−*06*	*7.00e*−*07*
	LLLLV	8.92e−05	3.10e−06	4.70e−06
krtm-sheet	TGTLE	1.05e−05	5.40e−06	6.30e−06
tm-sheet	PTLDL^#^	0.0001878	2.77e−05	<*1.00e*−*08*
	LYGKV^#^	0.0001610	<*1.00e*−*08*	<*1.00e*−*08*
	SASAG^#^	0.0001342	*1.40e*−*06*	<*1.00e*−*08*
	RQFNV	0.0001342	*2.20e*−*06*	*1.40e*−*06*

Given are the sequence pool name (column 1) and sequence with the highest frequency of occurrence (column 2); the frequency of occurrence (FO) of this peptide in the according pool (column 3), the FO in the pool containing all sequences except the one of the analyzed pool (column 4), the FO in the pool containing all sequences generated by the same strategy (GBSS) excluding sequences of the analyzed pool (column 5). Italic shows peptides with at least 50-fold higher frequency with respect to all (column 4) or peptides of the same strategy (column 5). Hashtag after the pattern indicate 500-fold higher frequency in at least column 4 or column 5

### Factors influencing pool separation

**Fig. 7:**
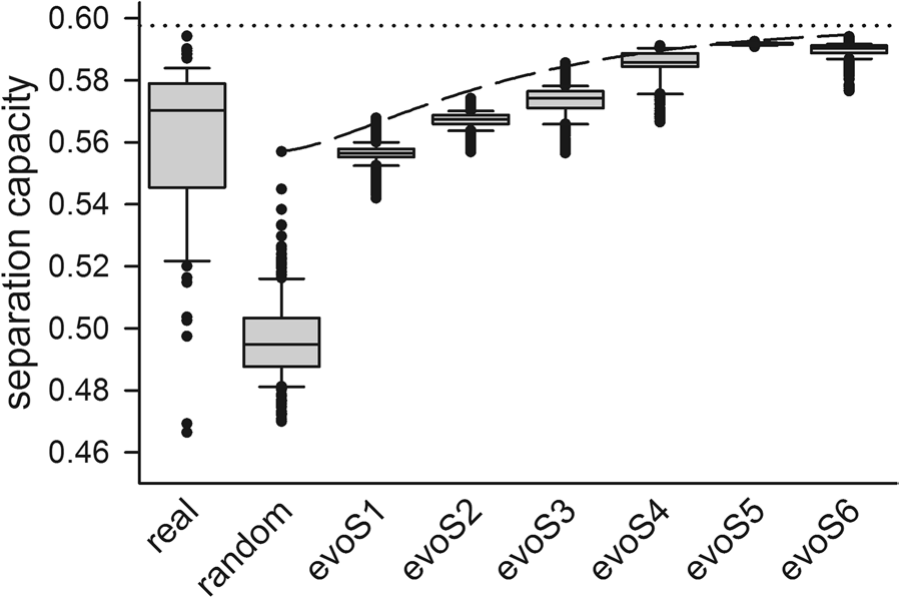
Separation capacity using evolution of random in silico scales. Shown is the *box* plot of separation capacity distribution of the 98 real hydrophobicity scales (real), the 200 randomly created scales (random, see “Methods” section) and the six in silico evolution steps (evoS1 to evoS6). The evolutionary optimization of the evolutionary approach was analyzed for the best performing scale identified after each step (*dashed line*) and the predicted plateau of 0.588 is shown as *dotted line*

The variation of separation capacity of hydrophobicity scales (Fig. 3) prompted the analysis of the impact of individual amino acid values. However, we did not realize any correlation between specific distribution of values for individual amino acid within the individual scales and the scale performance based on the 98 known hydrophobicity scales. As an alternative approach we created random hydrophobicity scales based on the 98 already known ones (Tables 3, 4). At first, the maximum and minimum amino acid values of the 98 real scales were used as interval to create 200 random hydrophobicity scales by assignment of a random value to each individual amino acid. Subsequently, several rounds of in silico evolution were performed to improve the separation capacity for the five different structural sequence pools (Fig. 7).

After six rounds of in silico evolution the created random hydrophobicity scales reached a separation threshold of 0.6, which is comparable to the separation potential of the best performing hydrophobicity scale. This suggests that a limit of the potential of amino acid scales for the separation of structural sequence pools exists by 0.6. Furthermore, we realized during the evolution of the hydrophobicity scales that the value of some amino acids had greater positive or negative influence on the separation capacity like others.

After establishing the evolutionary scale, we aimed at an understanding which property of a scale has an impact on its separation capacity. At first, we tested whether the general order of amino acids with respect to their hydrophobicity value is important. We realized that it is not the overall order of the amino acid hydrophobicity values that influences the performance of the hydrophobicity scale (Additional file 7: Fig. S2). At second we analyzed whether the value of specific amino acids dominate the separation capacity of a scale. We realized high S values for hydrophobicity scales sharing rather comparable hydrophobicity values for Gln, His, Gly, Ser or Arg to the evolved scale or for scales with hydrophobicity values for Cys, Met, Lys, Val or Ile distinct from the evolved scale (Additional file 8: Fig. S3). Thus, the hydrophobicity value of some amino acids like Gln, His, Gly, Ser or Arg might be more important for the separation capacity of the scales than others.

**Fig. 8:**
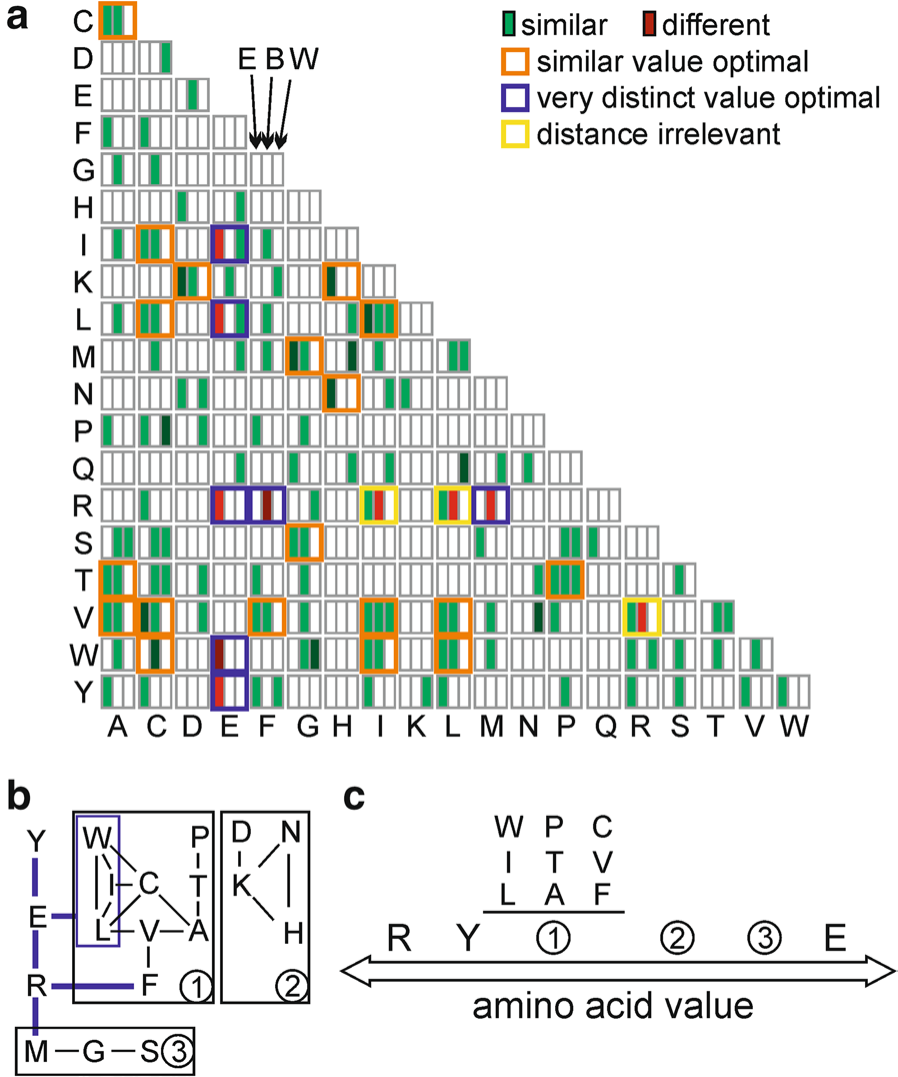
Distance of amino acid value in hydrophobicity scales. **a** Calculated was absolute difference between the values of two amino acids for the best performing evolutionary derived scale (*first box*), of scale 14 (highest S value, *second box*) and of scale 40 (lowest S value, *third box*) after normalization of the scales to (X-min)/(max–min). *Green boxes* mark distances below 0.1, *dark green boxes* below 0.01, *red boxes* distance above 0.9 and *dark red boxes* distance above 0.99. Combination framed in *orange* mark amino acids for which values should be rather similar as concluded from the low difference in the best performing and the evolutionary evolved scale, *blue frames* mark amino acid combinations for which values should be rather different as concluded from the low difference in the best performing and the evolutionary evolved scale and *yellow frames* mark amino acid combination for which the value difference is irrelevant. **b** The clusters with comparable (*black lines*) or distinct amino acids values (*blue lines*) are shown. **c** The *arrow* indicates the distance of amino acid values that should be present in a good performing scale

Thirdly, we asked whether cluster of amino acids with comparable or rather distinct values exist within one scale, which lead to high S values. To this end we analyzed the difference between hydrophobicity values of amino acids of individual scales, namely of the in silico evolved scale, the experimental hydrophobicity scale with highest (best) and the scale lowest (worst) S value, respectively. Each scale was normalized as such that the highest hydrophobicity value within the scale was set to one and the lowest hydrophobicity value to zero. Subsequently the difference of hydrophobicity values of the amino acids of one scale was calculated (Additional file 9: Table S6). Finally we analyzed whether a pair of amino acids shows a very small (<0.1, Fig. 8a green field) or very large (>0.9, Fig. 8a, red field) difference of the hydrophobicity value within each of the three scales. Finally, we inspected which pairs of amino acids show a similar low difference in the experimental scale with highest S value and the evolutionary scale (Fig. 8a, orange frame). In addition, we selected amino acids pairs with very different hydrophobicity value at least in one of the two scales (Fig. 8a, blue frame) and such pairs where the difference was small in one and large in the other one of these two scales (Fig. 8a, yellow frame).

Inspecting the information we realized that a large difference of the hydrophobicity values for glutamate and arginine to each other exists. In addition, the hydrophobicity value of glutamate is most distant to the hydrophobicity values of tyrosine, tryptophan, leucine and isoleucine, respectively Fig. 8a, blue frame). The hydrophobicity value of arginine is distant to the value of phenylalanine and methionine (Fig. 8a, blue frame). In turn, three distinct clusters of amino acids with comparable hydrophobicity values become obvious (Fig. 8a, orange frames). Considering all pairs one can draw relations of the hydrophobicity values within these clusters. Interestingly, the hydrophobicity values of cluster three are most distant form arginine (Fig. 8b), while the hydrophobicity values of cluster one are most distant to glutamate. However, these clusters do not correlate with the amino acid pattern detected for the specific sequence pools (Tables 6, 7) and moreover, they do not necessarily represent the physicochemical properties of the amino acids.

## Conclusion

We demonstrate that most of the hydrophobicity scales reach the same level of peptide separation capacity (Figs. 3, 4) and thereby, the method by which the scale was generated has no direct influence on clustering or separation capacity (Figs. 1, 3). Nevertheless, if at all we realized that the scale 14 defined by Naderi-Manesh developed in 2001 [40] performs somewhat better than the other hydrophobicity scales. We propose a rule of thumb for experimentalists that aim to use a hydrophobicity scale for identification of peptides with transmembrane segments from a pool of peptides. The hydrophobicity value of arginine and tyrosine should be most distant from the value of glutamate, while the hydrophobicity values of Asn, Asp, His, Lys should be in the center of the scale (Fig. 8c). We further observed that separation of sequence pools defined by known secondary structures is more likely than separation of sequence pools with a combination of secondary structures derived from in silico digestion (Figs. 3, 4), but the tryptic digested sequence pools with helical and strand content or transmembrane ß-strand or α-helix content are best separable from the other pools (Fig. 4). Nevertheless, we realized a threshold of S = 0.6, irrespective of the nature of the scale, which is supported by an in silico approach to optimize the scale (Fig. 7). In turn, the separation capacity depends on the number of parameter calculated (Additional file 3: Fig. S1), although we realized that the alternating hydrophobicity has the lowest capacity for sequence pool separation (Fig. 5). Remarkably, we observed that detection of ß-strands in peptides can be supported by the detection of penta-peptides (Fig. 6) because such peptides have been detected in the structural pool and in the pools generated by simulated tryptic digest (Table 7). Similarly, amino acid patterns specific for the transmembrane ß-strands (Tables 6, 7) or largely random content (Table 7) have been observed. In turn, for pools mainly consisting of helical structures only one specific penta-peptide for soluble (s-helix) and transmembrane (krtm-helix) α-helices could be detected (Table 7). Summarizing, the quality of separation of sequence pools depends rather on the parameter used for calculation than on the scale used and can be supported by the search for specific amino acid pattern.

## Methods

### Hydrophobicity scales

98 hydrophobicity scales (Tables 3, 4)—16 are only reversed algebraic figures of other scales in the set were extracted from three different sources (http://www.genome.jp/aaindex/ [30]; http://split4.pmfst.hr/split/scales.html [22]; http://web.expasy.org/protscale/ [31]). The path of hydrophobicity scales development is given in Additional file 10: Fig. S4.

### Hydrophobicity parameter

Five different hydrophobicity parameters (Table 5) were used to analyze their influence on the separation capacity. For all of those hydrophobicity parameters we used contrary pairs (max. and min.) of the parameters to look for potential differences. The EBBS [16] should be able to detect β-sheets, whereas the alternating hydrophobicity [14, 15] should be more specific to detect transmembrane β-sheets. The hydrophobicity moment α and β [13] were used to identify α-helices and β-sheet in general. The last parameter was the average hydrophobicity, which had no preferentially detectable secondary structure so far.

### Structure pools

The known secondary structure pools (Tables 1, 2) were extracted from the ASTRAL40 database [32] and differed in random coil (random), cytosolic β-sheets (s-sheet), cytosolic α-helix (s-helix), transmembrane β-sheet (tm-sheet) and transmembrane α-helix (tm-helix). Further, we implemented an in silico tryptic digest approach to split sequences after Lysine (K) and Arginine (R) of the whole ASTRAL database and classified the peptide fragments concerning their secondary structures. These were divided in fragments containing a (i) continuous dominating SSE (dc), (ii) discontinuous dominating SSE (dd), (iii) no dominating SSE but only two different structures (no-), (iv) all three secondary structures (all) or (v) transmembrane sheet or helix fragments (krtm-).

### Pool separation via hydrophobicity scales and parameter

#### Cloud algorithm

The algorithm to calculate the cloud is a two-step approach. All single peptide sequences of a specific secondary structure dissection pool (Table 1) or in silico K/R-digestion pool (Table 2) were used as input to calculate the cloud. Thereby, each peptide is represented by an n-dimensional vector (hyper volume; convex cover), where the values of the different hydrophobicity parameters (n ≤ 5 dimensions; represented by the minimum or maximum value using a specific hydrophobicity parameter from Table 5) calculated based on a specific hydrophobicity scale (Tables 3, 4) are the components of this vector.

(I) The initial cloud is calculated based on a randomly chosen as subset of ~30 points (peptides defined by vectors). Then, the cloud is expanded until each point is considered. In general, the algorithm calculates all distances and directions within the n-dimensional space between all given points (peptides) and tests if these sites are valid. A site is valid if all points of the entire cloud follow the direction of the hypersurface. By this it is determined if an added point lies within the so far calculated cloud.
(II) The existing cloud is expanded point by point to determine the cloud by a set of sites between points. After each point the set of sites is updated by the procedure (i) and all remaining points are tested if these points are inner points or putative boundary points.
(III) The final cloud volume is calculated based on the outer sites between the boundary points in the n dimensional space that form a convex envelope. All points are placed inside the cloud (inner points) or on the convex envelope (boundary points).

#### Separation calculation

All peptides of two structure pools in a given scenario were used to calculate the heuristic hypervolumes of each pool, respectively, defined by the hypersurface via a pipeline. A scenario is defined by the used number of dimensions represented by the selected hydrophobicity parameters, the selection of the hydrophobicity scale and which two structure pools are used for comparison. The number of the points (peptides) positioned by the according vector within the clouds was counted as well as the points within the overlap of both cloud volumes.

#### Convex envelope

We remove the boundary points (petides) of both structure pools building the convex envelope to avoid big volumes of the clouds based on outliers. We analyzed the volume and number of peptides per structure pool for all combinations with n = 5 dimensions and calculate the loss of peptides and loss of volume in percentage. Due to the high amount of combinations per structure pool (defined by number of hydrophobicity parameter and number of hydrophobicity scales) we calculate the minimum, maximum and average of volume and peptide reduction removing the boundary points (Additional file 11: Table S7). In average, this procedure leads to an elimination of 6.8 % peptide sequences of the structure pool, but a decrease of the according volume of 44.6 %. By that, removal of putative outlier cause on average a sevenfold increase of the volume per peptide. For pools with low amount of peptides (krtm-sheet, krtm-helix, no-random) the increase of the volume per peptide is lower, namely in the order of twofold. Nevertheless, this increase of density justifies the procedure.

### Hydrophobicity scale clustering

For the hydrophobicity scale clustering the dissimilarity of the different pairs of hydrophobicity values for each amino acid was calculated. This was done by using autocorrelation between all pairs of the 98 different hydrophobicity scales. Afterwards, the Pearson correlation values were normalized to get the dissimilarity and used by MEGA6 [34] to create an UPGMA tree of the dissimilarity. The clustering of the hydrophobicity scales was done by determining a threshold of 0.05 (5 %) for dissimilarity to split the tree in groups.

### Amino acid pattern search

For the amino acid pattern search the different structure pools were used. First, the peptide fragments were analyzed for all occurring amino acid patterns of a specific length based on a Markov chain algorithm of the MEME and MAST suite package (fasta-get-markov) [43]. The algorithm estimates a Markov model from a FASTA file of sequences with previous filtering of ambiguous characters. For example a peptide of four amino acids in length has a conditional probability that one amino acid follows the other amino acid given a specific pool of peptide sequences. So the Markov chain allows the calculation of the transition probability from one state to another state and by this determines the probability of an amino acid occurring in an amino acid peptide of a certain length of a specific pool of peptides. In this approach all possible patterns were detected in the peptides starting from a pattern length of one and incrementing by all different 20 possibilities for each amino acid. The occurrence of the different pattern was normalized to one and compared to the occurrence of the other structure pools to determine the pairwise difference between the pools to detect pool specific pattern of specific length. Furthermore, we performed multiple testing with our identified pattern of length four and five amino acids. We used the Fisher exact test to calculate p values examining the significance of the contingency between occurrences of a specific pattern in relation to a specific structure pool. As reference we pooled all 17 structure pools together. To overcome artificial errors using multiple times the fisher exact test we used as post hoc test Benjamini/Hochberg false discovery rate (fdr) multiple test correction to adjust our p values (Additional file 5: Table S4, Additional file 6: Table S5, p values). All amino acid pattern of length four (Table 6) and five (Table 7) with an adjusted p value below α = 0.05 were marked in bold.

### In silico creation of random hydrophobicity scales

The generation of in silico hydrophobicity scales is based on the minimum and maximum hydrophobicity values extracted out of the 98 analyzed hydrophobicity scales, which were determined as borders for the interval. We used five structure pools to calculate the separation capacity score (dd-sheet, dd-helix, dd-random, krtm-sheet, krtm-helix). Two hundred random hydrophobicity scales were created. Based on the best in silico random hydrophobicity scale of the previous steps 2000 scales were created; 100 per amino acid. Half of the hydrophobicity scales per amino acid changed the hydrophobicity value of the single amino acid in the positive [0.001:5] and negative [−0.001:−5] interval (evo1 and evo2). In the following in silico evolution steps (evo3 to evo5) the top 100 newly generated hydrophobicity scales with best performance were analyzed to filter for amino acids which have an influence on the separation capsacity. Only these amino acids were changed in the evo steps evo3 to evo5 to analyze their influence. For evo3 100 hydrophobicity scales per amino acid were created changing within the interval [0.001:10] for E and Y and [−0.001:−10] for A, H, F and L. For evo4 200 hydrophobicity scales per amino acid were created changing within the interval [0.001:20] for E and [−0.001:−20] for A and H. In evo5 400 hydrophobicity scales were created changing within the interval [0.001:40] for E. Finally, in evo6 1000 random hydrophobicity scales based on the best scale of evo5 were created. For each amino acid 25 hydrophobicity scales were created changing within the positive [0.001:5] and 25 scales were created changing within the negative [−0.001:−5] interval.

## Additional files

10.1186/s40659-016-0092-5 Hydrophobicity scale values. Given is the index of the hydrophobicity scale (column 1), the year of publication (column 2), the authors (column 3), the name of the scale (column 4), the website for downloading the scale (column 5) and the hydrophobicity values for each amino acid (columns 6–25).
10.1186/s40659-016-0092-5 Correlation of hydrophobicity scales. Given is the correlation matrix of the 98 hydrophobicity scales based on the hydrophobicity values of the 20 amino acids. The name of the scale (column 1 and line 1) as well as the index of the scales (column 2 and line 2) is given.
10.1186/s40659-016-0092-5 Separation capacity using different parameter dimensions. Shown is the separation capacity using a different amount of hydrophobicity parameter (3–5 dimensions, see legend in B); (A) shows the separation of tryptic digested pools, (B) of secondary structure pools and (C) of all mixed pools. The 98 hydrophobicity scales on the X-axis are sorted descending the separation capacity in (B). A separation value of 0 means full-overlap of the pools and 1 means no overlap.
10.1186/s40659-016-0092-5 Amino acid composition in sequence pools. Given are the sequence pool (column 1) and the occurrence of each amino acid in the given pools normalized to 1.
10.1186/s40659-016-0092-5 Amino acid pattern of length 4. Given is the sequence pool 1 (column 1) and sequence pool 2 (column 2), the separation capacity between pools based on hydrophobicity (column 3), the maximal difference in the frequency of occurrence (FO) pattern value (column 4), the minimal difference in the FO pattern value (column 5), the number of overrepresented pattern from pool1 in contrast to pool2 (column 6) and the top 5 of identified pattern of length 4.
10.1186/s40659-016-0092-5 Pool specific amino acid pattern of length 5. Given is the sequence pool 1 (column 1) and sequence pool 2 (column 2), the separation capacity between pools based on hydrophobicity (column 3), the maximal difference in the frequency of occurrence (FO) pattern value (column 4), the minimal difference in the FO pattern value (column 5), the number of overrepresented pattern from pool1 in contrast to pool2 (column 6) and the top 5 of identified pattern of length 5.
10.1186/s40659-016-0092-5 Normalized amino acid hydrophobicity values of evolved scale. Shown is the normalized hydrophobicity value of all 20 amino acids for the best real (scale 28), worst real (scale 40) corresponding to the five selected sequence pools and in silico evolved hydrophobicity scale as radar plot. Evo scale green squares; best real scale black circles; worst real scale red triangles.
10.1186/s40659-016-0092-5 Correlation of amino acid hydrophobicity distance to evolution scale and separation capacity score of real hydrophobicity scale. Shown is the correlation via linear fit between the separation capacity for the 98 real hydrophobicity scales and the distance of hydrophobicity value of a single amino acid to the in silico evolved scale. The single amino acids are distributed to four graphs (A–D) concerning their slope of the individual linear fit. (A) Raising slope red; (B) slightly raising slope blue; (C) no raising slope black; (D) falling slope green.
10.1186/s40659-016-0092-5 Distance of amino acids hydrophobicity values to evolved random scale. Given is the scale identifier (column 1) and scale separation capacity (column 2). Furthermore, for each amino acid (columns 3 to 22) the distance of the normalized hydrophobicity value to the evolved hydrophobicity scale value is shown.
10.1186/s40659-016-0092-5 Organigram of improved hydrophobicity scales. Shown is the relation of hydrophobicity scales with respect to their origin. The dependencies (shown by directed graph) are based on exhaustive literature search. The green marked hydrophobicity scales were included in our study and the red ones not.
10.1186/s40659-016-0092-5 Influence of convex envelope on volume and number of peptides. Represented is the reduction of volume and number of peptides per structure pool (column 1; number of all peptides within the pool, column 2) in percentage for all scenarios with n = 5 dimensions in average (columns 3, 4), in minimum (columns 5, 6) and in maximum (columns 7, 8).
