# Supplementary figures and images for: 50 years of amino acid hydrophobicity scales: revisiting the capacity for peptide classification
Source: Biol Res. 2016 Jul 4;49:31. doi: 10.1186/s40659-016-0092-5 (PMC4932767; doi:10.1186/s40659-016-0092-5)

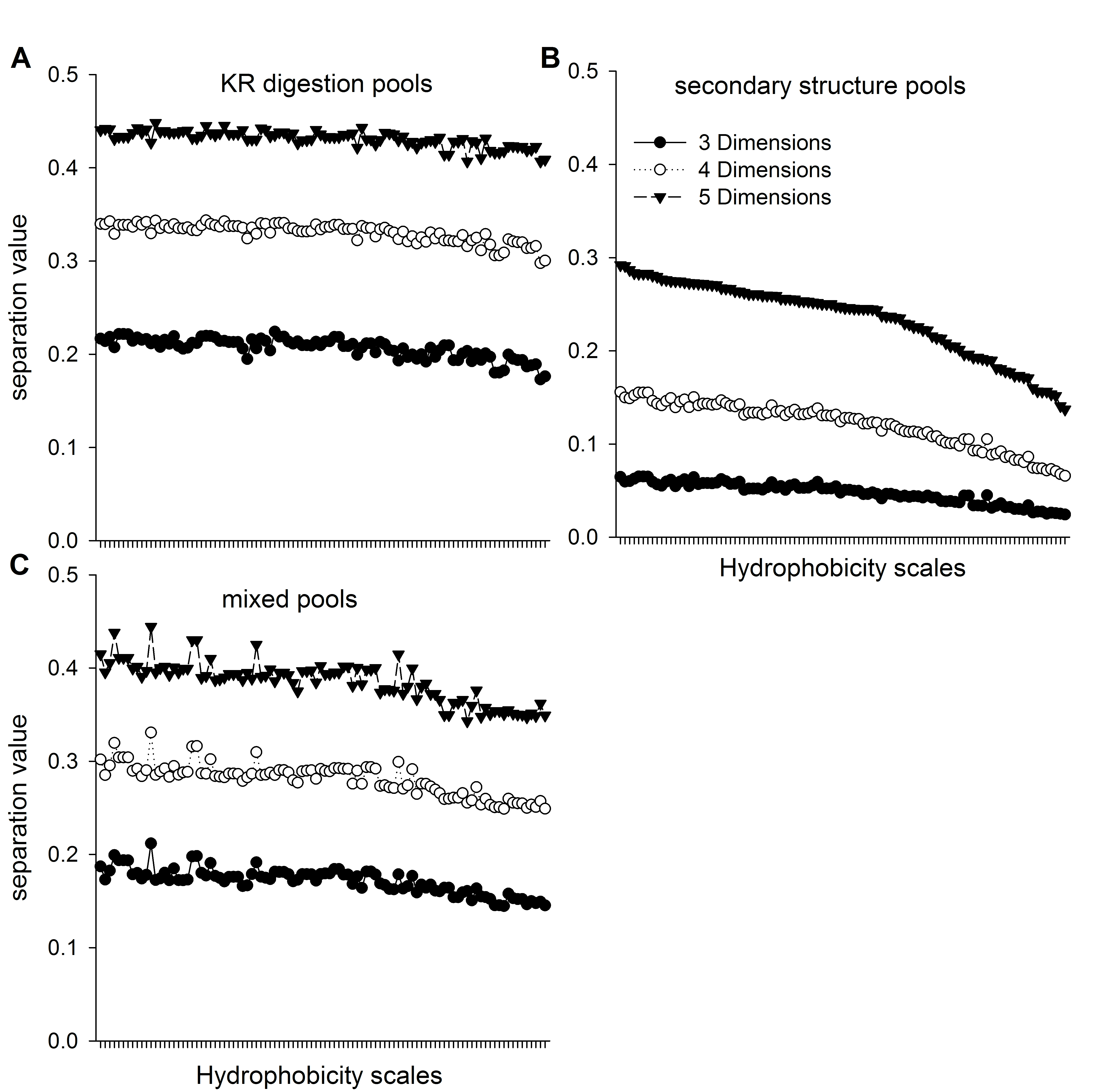

Supplement: Supplementary file 3 — 10.1186/s40659-016-0092-5 Separation capacity using different parameter dimensions. Shown is the separation capacity using a different amount of hydrophobicity parameter (3–5 dimensions, see legend in B); (A) shows the separation of tryptic digested pools, (B) of secondary structure pools and (C) of all mixed pools. The 98 hydrophobicity scales on the X-axis are sorted descending the separation capacity in (B). A separation value of 0 means full-overlap of the pools and 1 means no overlap. [file 40659_2016_92_MOESM3_ESM.tiff]

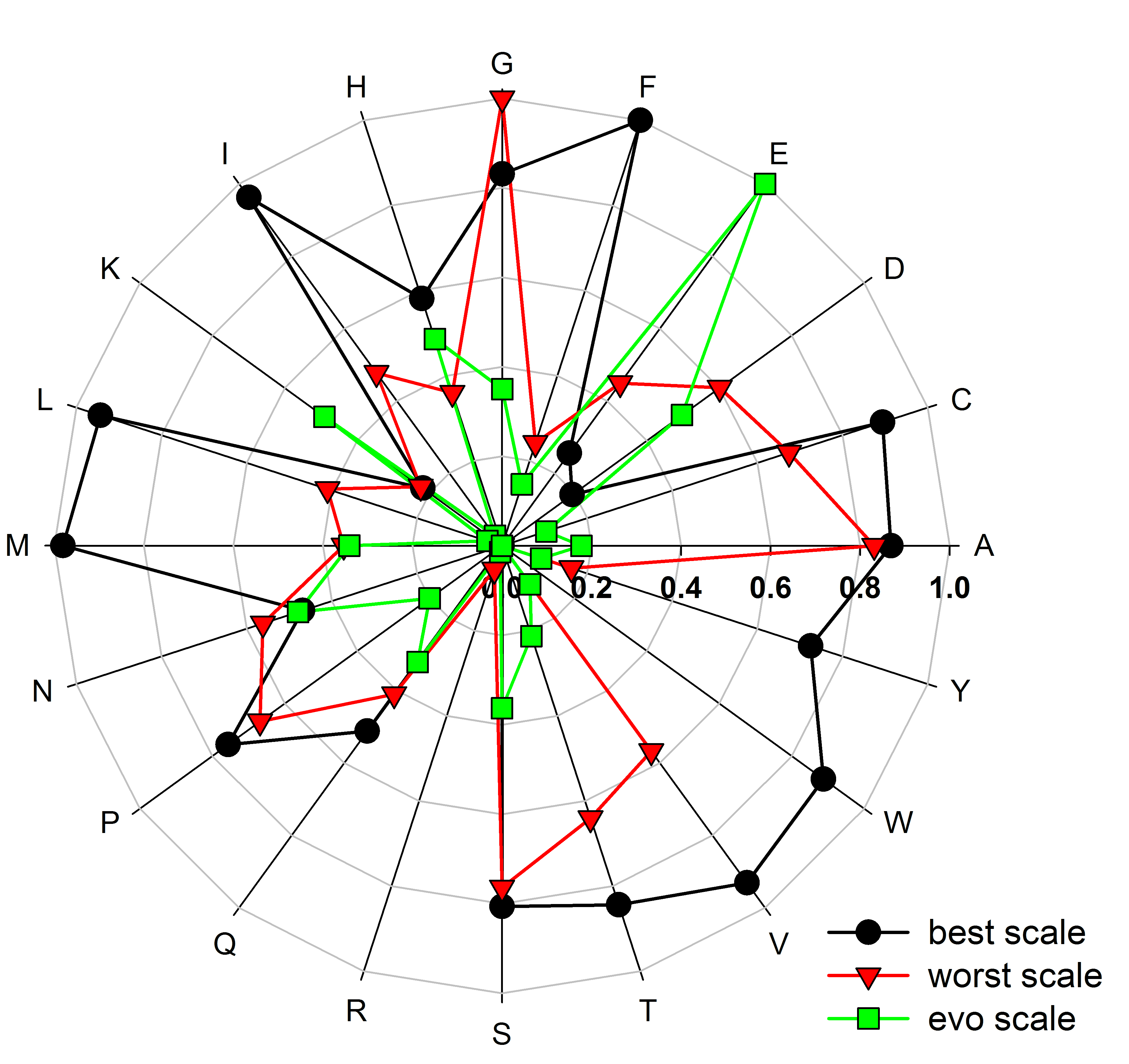

Supplement: Supplementary file 7 — 10.1186/s40659-016-0092-5 Normalized amino acid hydrophobicity values of evolved scale. Shown is the normalized hydrophobicity value of all 20 amino acids for the best real (scale 28), worst real (scale 40) corresponding to the five selected sequence pools and in silico evolved hydrophobicity scale as radar plot. Evo scale green squares; best real scale black circles; worst real scale red triangles. [file 40659_2016_92_MOESM7_ESM.tiff]

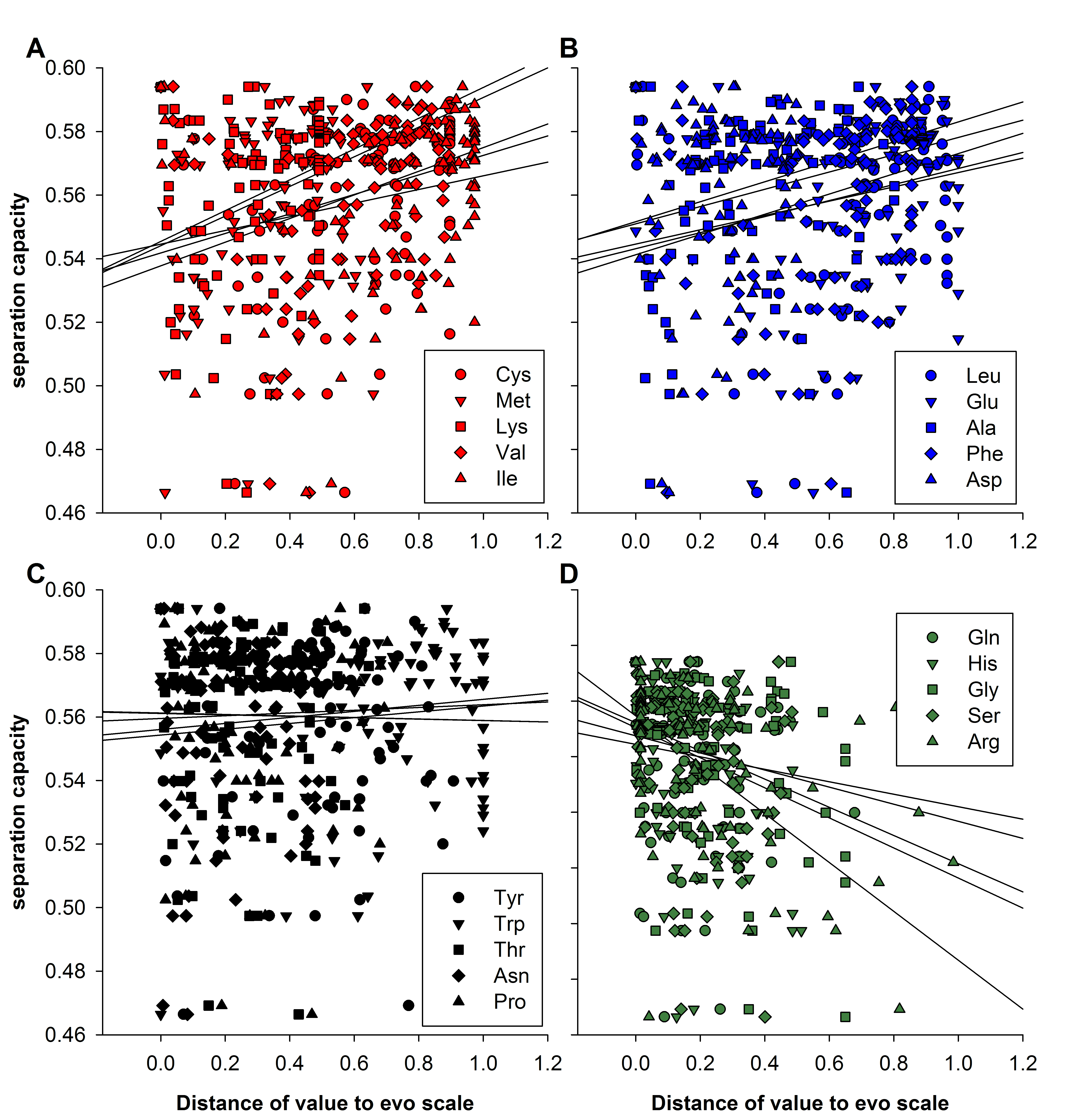

Supplement: Supplementary file 8 — 10.1186/s40659-016-0092-5 Correlation of amino acid hydrophobicity distance to evolution scale and separation capacity score of real hydrophobicity scale. Shown is the correlation via linear fit between the separation capacity for the 98 real hydrophobicity scales and the distance of hydrophobicity value of a single amino acid to the in silico evolved scale. The single amino acids are distributed to four graphs (A–D) concerning their slope of the individual linear fit. (A) Raising slope red; (B) slightly raising slope blue; (C) no raising slope black; (D) falling slope green. [file 40659_2016_92_MOESM8_ESM.tiff]

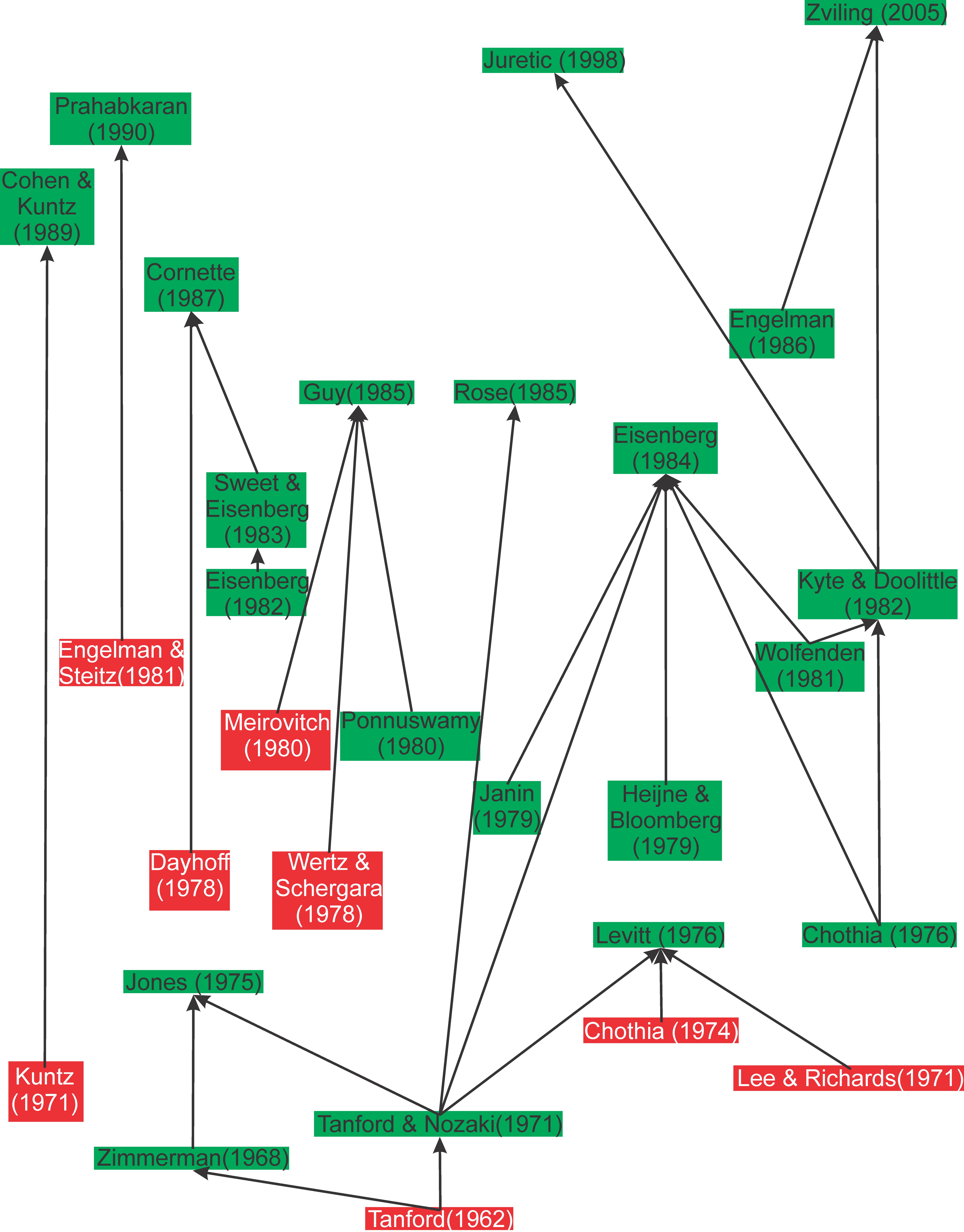

Supplement: Supplementary file 10 — 10.1186/s40659-016-0092-5 Organigram of improved hydrophobicity scales. Shown is the relation of hydrophobicity scales with respect to their origin. The dependencies (shown by directed graph) are based on exhaustive literature search. The green marked hydrophobicity scales were included in our study and the red ones not. [file 40659_2016_92_MOESM10_ESM.tiff]
